# Self-organizing Mechanism for Development of Space-filling Neuronal Dendrites

**DOI:** 10.1371/journal.pcbi.0030212

**Published:** 2007-11-16

**Authors:** Kaoru Sugimura, Kohei Shimono, Tadashi Uemura, Atsushi Mochizuki

**Affiliations:** 1 Graduate School of Biostudies, Kyoto University, Kyoto, Japan; 2 Department of Science, Kyoto University, Kyoto, Japan; 3 Division of Theoretical Biology, National Institute for Basic Biology, Okazaki, Japan; University College London, United Kingdom

## Abstract

Neurons develop distinctive dendritic morphologies to receive and process information. Previous experiments showed that competitive dendro-dendritic interactions play critical roles in shaping dendrites of the space-filling type, which uniformly cover their receptive field. We incorporated this finding in constructing a new mathematical model, in which reaction dynamics of two chemicals (activator and suppressor) are coupled to neuronal dendrite growth. Our numerical analysis determined the conditions for dendritic branching and suggested that the self-organizing property of the proposed system can underlie dendritogenesis. Furthermore, we found a clear correlation between dendrite shape and the distribution of the activator, thus providing a morphological criterion to predict the in vivo distribution of the hypothetical molecular complexes responsible for dendrite elongation and branching.

## Author Summary

Neurons elaborate two types of neuronal extensions. One is axon, which sends outputs to other neurons. Another is dendrite, which is specialized for receiving and processing synaptic or sensory inputs. Like elaborated branches of trees, the shape of dendrites is quite variable from one type to another, and different dendritic geometry contributes to differential informational processing and computation. For instance, neurons of the space-filling type (e.g., retinal ganglion cells) fill in an open space to pick up spatial information from every corner of their receptive field. Therefore, dendrite development is one of the representative examples of the emergence of function through morphogenesis. Previous experiments including ours showed that competitive dendro-dendritic interactions play critical roles in shaping dendrites of the space-filling type. In the present study, we incorporated this finding in constructing a new mathematical model, in which reaction dynamics of chemicals are coupled to neuronal dendrite growth. Our numerical analysis suggested that self-organizing property of the proposed system underlies formation of space-filling dendrites. Furthermore, we provided a morphological criterion to predict the in vivo distribution of the hypothetical molecular complexes responsible for dendrite elongation and branching. We have now found a substantial number of molecules involved in dendrite development, thus it is timely to discuss the prediction from this work.

## Introduction

One of the primary interests in developmental biology is the emergence of function through morphogenesis. Morphological diversity of dendrites and its impact on neuronal computation perfectly represents the importance of this problem: shapes of dendrites are highly variable from one neuronal type to another, and it has been suggested that this diversity supports differential processing of information in each type of neuron [1–3]. Therefore, patterning of neuronal class-specific dendrites is a process to produce shapes that realizes the physiological functions of neurons. Recent advances in genetic manipulation at the single-cell level enabled us to identify genes whose loss of function affects neuronal morphology (reviewed in [4–6]); however, we are far from formulating an overall picture of the underlying mechanism of pattern formation.

**Figure 1:**
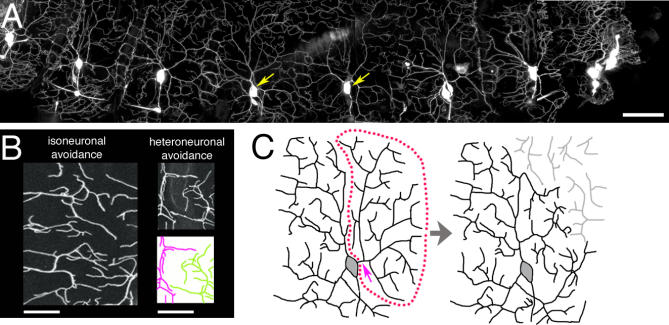
Competitive Interactions between Dendrites Mediate Isoneuronal Avoidance and Tiling (A) An image of *Drosophila* larva of *NP7028 UAS-mCD8::GFP* [11]. Class IV da neurons ddaC (arrows) were visualized with GFP. Dendrites of class IV da neurons almost completely cover the body wall. (B) A high-power image of class IV da neurons at single dendrite resolution (left). Dendrites from the left segment are colored purple; and those from the right segment, green (right). Dendrites of the same class IV da neuron come very close, but hardly overlap each other (isoneuronal avoidance); in addition, minimal overlap was seen between dendrites of neighboring neurons (heteroneuronal avoidance or tiling). (C) Schematic drawing of a filling-in response (adapted from 12). Left: Branches enclosed by the dotted line were severed by laser irradiation (arrow). Right: Neighboring dendrites filled in the open space that had been covered by the detached branches and space-filling pattern was regenerated. Black: dendrites of the operated cell. Gray: dendrites of the neighboring cell. This experiment clarifies an essential role of inhibitory dendro-dendritic interactions in isoneuronal avoidance and tiling. Bar, 50 μm for “A” and 20 μm for “B.”

Among various classes of dendrites is the “space-filling” type, which uniformly covers its receptive field. The concept of space-filling was introduced by Fiala and Harris [7], and we use this term with a slightly different meaning here. Neurons elaborating space-filling dendrites are found in various parts of nervous system, including retinal ganglion cells [8], trigeminal ganglion cells [9], Purkinje cells (Figure 8B) [10], and *Drosophila* class IV dendritic arborization (da) neurons (Figure 1) [11–14]. The space-filling type looks very complex morphologically, but can be regarded as being simple in their isotropic features and in their two-dimensionality. Most importantly, it shows distinctive spatial regulation of pattern formation: for instance, dendritic branches of *Drosophila* class IV da neurons avoid dendrites of the same cell and those of neighboring class IV cells, which allows complete, but minimal overlapping, innervation of the body wall (designated as isoneuronal avoidance and tiling) (Figure 1A and 1B) [11,13–15]. Our previous experiment together with studies by others demonstrated that competitive dendro-dendritic interaction underlies tiling, as shown by the fact that the da neurons reaccomplish tiling in response to ablation of adjacent neurons of the same class or to severing of their branches (Figure 1C) [11,14]. It should be noted that the qualitatively same inhibitory dendro-dendritic interaction is working between the adjacent neurons of the same type as well as between dendrites of the same neurons.

There are two types of the proposed mechanisms that support this repulsive behavior of dendrites: one is contact-dependent retraction of dendrites and the other is repulsion of dendrites via diffusive suppressors. The contact-dependent mechanism seems insufficient to a clear field splitting, because as far as dendrites do not make contacts (by passing under other dendrites, for example) they can invade neighboring territories. Moreover, time-lapse analysis showed that dendrites make a turn before they are about to cross nearby branches [16]. So we prefer diffusive signaling to a contact-dependent one. Similar mechanisms have been suggested to work in other model systems as well [9,17,18]. With all available information taken together, we considered the space-filling dendrite to be an ideally suited one for us to start modeling, due to the simplicity of its patterning and the experimentally verified mechanism of the pattern formation.

A number of mathematical models for neurite formation were previously proposed; and most of them assumed that dendrite development is a consequence of stochastic sprouting and subsequent growth arrest [19–22]. Different forms of branching functions were postulated and modified so that calculated dendrograms would fit dendritic arbors of real neurons in a quantitative manner. Those studies were descriptive and did not provide a comprehensive mechanism of pattern formation. In this study, we developed a new class of mathematical model for neurite formation to approach a principle of development of the space-filling dendrites. In our neurite growth model that is based on the aforementioned inhibitory dendro-dendritic interaction, various aspects of pattern formation, e.g., extension, orientation of growth, and branching of dendrites, are represented in a single framework. Computer simulation showed that our model develops dendritic extension and branching autonomously; furthermore, numerical analysis determined the conditions for dendritic growth.

## Results

### Cell Compartment Model for Dendritic Growth

**Figure 2:**
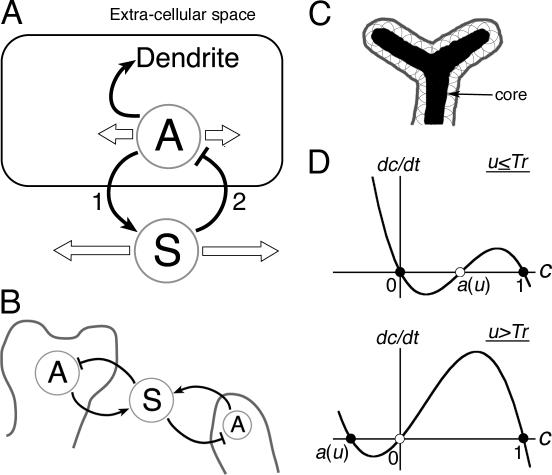
Schematic Representation of the Cell Compartment Model (A,B) An activator–suppressor system. Intracellular activator promotes growth of dendrite and produces the suppressor or accelerates secretion of the suppressor from intracellular organelles (“1” in (A)). On the other hand, the suppressor is secreted from the cell and diffuses in extracellular compartments. Binding of its receptor on the plasma membrane triggers signaling to inhibit synthesis of the activator (“2” in (A)). These reactions underlie inhibitory dendro-dendritic interactions (B). (C) Black: core of the cell; dark gray: cell boundary. The cell compartment is represented by collective circular domains around the core with radius *R* (gray circles). (D) Dynamics of core of the cell (*c*). The activator promotes cell growth when its concentration is higher than threshold (*Tr*). *a*(*u*) = 0.49 (*u* ≤ *Tr*) or *a*(*u*) = 0.49 − 2.5(*u* – *Tr*) (*u* > *Tr*). Upper graph: Both *c* = 0 and *c* = 1 are stable equilibrium points. Lower graph: When *a*(*u*) < 0, *c* = *a*(*u*) and *c* = 1 are stable equilibrium points and *c* = 0 becomes an unstable equilibrium point. Very small positive noise was added to *c*, so *c* → 1 quickly. These settings make it possible to store the history of growth of *c*, because *c* = 1 is a stable equilibrium point all the time.

As mentioned above, two-dimensionality is a characteristic of space-filling dendrites; thus we built our model in the 2D space, dividing the 2D space into two distinct compartments (Figure 2A), i.e., the compartment occupied by neurons (designated as the cell compartment or the cell region hereafter) and the extracellular compartment. This model is referred to as the “cell compartment model.” We assumed that growth of the cell region, which shapes the dendritic trees, is regulated by a hypothetical intracellular chemical, i.e., the activator (Figure 2A). We set a restriction in terms of the movement of the activator so that it diffuses only the inside of cells. The activator promotes the growth of the cell compartment when its concentration is higher than threshold (*Tr* in Figure 2D). To account for the inhibitory dendro-dendritic interaction, we hypothesized another chemical, the suppressor. The suppressor is produced when the concentration of activator is high, i.e., it is produced at the actively growing region of dendrites. The suppressor acts to decrease the concentration of activator, but the concentration of activator can increase by its autocatalytic production where the activator is locally concentrated. The reaction between activator and suppressor is the so-called “activator-inhibitor type” [23,24] (“1” in Figure 2A). The activator-induced production of the suppressor can be realized by either local translation of suppressor-encoding mRNA in dendrites [25] or secretion of suppressor proteins from intracellular organelles. The suppressor is secreted from the cell, diffuses throughout the extracellular space, and then binding of the suppressor to its receptor drives intracellular signaling to repress the production of the activator (“2” in Figure 2A). So, the suppressor mediates long-range inhibitory interactions between dendrites (Figure 2B). These settings endow the system with feedback-loop regulation at two different levels: one is between the two chemicals and the other is between the dynamics of these chemicals and the expansion of the cell compartment. The latter consists of the following reciprocal interactions: the activator controls growth of the cell region and growth of the cell region determines where the activator can diffuse further.

### Model Formulation

Our model can be written as the following equations:

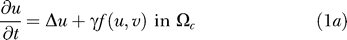


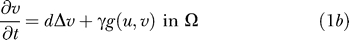


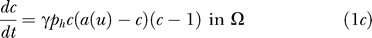
where *u* and *v* are concentrations of the activator and the suppressor, respectively. Note that these equations are already non-dimensionalized, so *d* is the ratio of the diffusion coefficient between the two substances (see the section “Original equations”). As we hypothesize that diffusion of the suppressor is faster than that of the activator, *d* is larger than 1 [26]. *c*(**x**, *t*) is a symbolic variable to indicate the “core” of the cell (Figure 2C and 2D). The biological correlates of *c* could be microtubules that support structural integrity of the cell. The right-hand side of Equation 1c indicates that the dynamics of the cell state is bi-stable and that the two steady states are 1 and 0, indicating “core” and “not core,” respectively, and *a*(*u*) is the switching point at which the growth behavior of *c* is flipped. *c* quickly reaches 1 when *u* is higher than threshold (*Tr*). The symbol Ω is the 2D real space, and **x** *_c_* denotes a point in Ω, where *c* is larger than 0.5. Ω*c*, which is the region of the cell in Ω, and is defined by using **x** *_c_* as follows: 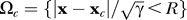 (*γ* is a rate constant to rescale time and space) [26]. *R* is the distance between core and the plasma membrane, and the cell compartment is represented by collective circular domains around the core with radius *R* (Figure 2C). We found that *R* = 0.004 realized the finest resolution of patterns, so we used this value of *R* throughout this study (see the section “*R* value” for details). Describing the cell growth as a rapid transition between bistable states is reminiscent of a way to solve moving boundary problems in phase-field models [27]. A difference between these models and ours is whether diffusion of the phase field is incorporated or not; a diffusion term does not appear in Equation 1c, because diffusion of the cell state is biologically unrealistic in this case.

*f*(*u*,*v*) and *g*(*u*,*v*) represent chemical reaction terms, where the partial derivatives satisfy the following conditions: 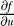 > 0 (autocatalytic production of the activator), 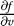 < 0 (inhibition of synthesis of the activator by the suppressor), 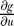 > 0 (production of the suppressor by the activator) and 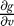 < 0 (concentration-dependent degradation of the suppressor). We used the following formulas for *f* and *g*:

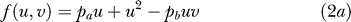


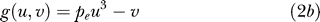



We assumed that the receptor is uniformly distributed over the dendritic surface and that the strength of the signaling follows the local concentration of the suppressor. We adopted the 0-fixed boundary condition for the activator at the cell boundary:

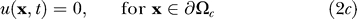



We used the periodic boundary for the other variables *v* and *c* at the boundary of the 2D square space to model the real 2D space Ω in numerical simulation.

### Autonomous Formation of Dendritic Patterns

**Figure 3:**
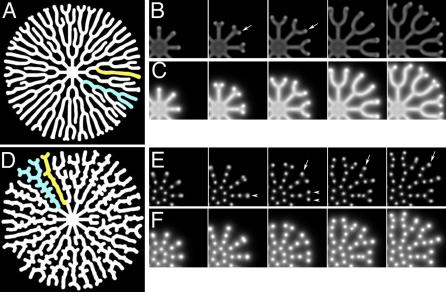
Distinctive Dendritic Patterns Obtained from the Computer Simulation of the Cell Compartment Model (A–C) and (D–F) Two distinct patterns obtained by using different parameter values in the activator–suppressor model. Whole images of dendritic trees (A,D), magnified images of the activator (B,E) and those of the suppressor (C,F). Examples of branch-poor arbors and branch-rich arbors were indicated in yellow and blue, respectively, (A) and (D). Density of the activator is relatively high at the terminal of each branch (arrows in “B”). Alternatively, dendrites elongate as new spots are generated (arrows in “E”) and bifurcate as spots undergo fission (arrowheads in “E”). Parameter values were *p_a_* = 0.9 and *p_e_* = 6.5 (A–C) and *p_a_* = 0.5 and *p_e_* = 2.6 (D–F). Other parameters were *p_b_* = 0.8, *d* = 30.0, *p_h_* = 1.0, *T_r_* = 1.0, A_max_ = 30.0, *R* = 0.004, and *γ* = 625. The grid size is 800 × 800, *dx* = 0.02, and *dt* = 1 × 10^−6^.

We numerically calculated the model given by Equations 1a–1c with reaction terms of Equations 2a and 2b (see Materials and Methods). Computer simulation showed that the cell compartment model could autonomously generate quite distinct dendritic patterns depending on the set of parameters employed (Figure 3). In each case where the model produced dendritic patterns, they were generated through repeated cycles of elongation and branching of dendrites (two examples are shown in Videos S1 and S3). With one set of parameters, smooth branches were formed, where neighboring branches aligned themselves nearly parallel to each other (Figure 3A). In such a cell, the distribution of the activator is continuous and mostly uniform, except for every branch terminal, where the density of the activator is relatively high (arrows in Figure 3B; Video S2). With a different set of parameters, the dendritic branches showed a more rugged morphology (Figure 3D). Stubby and non-aligned branches were formed, and the activator was distributed in a punctate manner in that cell (Figure 3E; Video S4). We call each punctum, where the activator was highly concentrated, a “spot.” Dendrites elongated by generating new spots (arrows in Figure 3E) and bifurcated when spots fissioned (arrowheads in Figure 3E). The suppressor was concentrated where the density of the activator was high, and it was distributed more broadly than the activator (Figure 3C and 3F). This distribution underlies long-range inhibitory interactions between neighboring dendrites. The interactions appeared to control whether or not dendrites would branch and in which direction dendrites would elongate. As a result, the branching frequency considerably varied among branchlets (compare yellow and blue arbors in Figure 3A and 3D), whereas the branch density was kept almost constant throughout the dendritic trees.

In a separately prepared manuscript, we addressed more biological issues such as tiling (Figure 1A and 1B) and regeneration in response to branch severing (Figure 1C). Branches of multiple neurons in our computer simulation, when they appeared in the same 2D space, avoided each other and accomplished tiling and isoneuronal avoidance. The neurons in our computer simulation were even able to reaccomplish tiling after local destruction of dendritic arbors exactly as *Drosophila* class IV da neurons do. Furthermore, modifications of our model enabled reproduction of a wide range of space-filling dendritic trees and even a non–space-filling type. Taken together, our model succeeded in qualitatively recapturing development of space-filling dendrites.

### Typical Behaviors of *u* and *v* at the Branch Terminals

**Figure 4:**
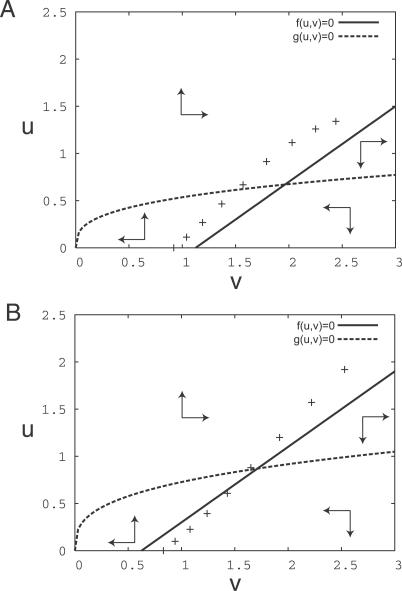
Typical Changes of *u* and *v* at the Tips of Branches (A,B) Representative data of the values of *u* and *v* were indicated on the phase plane, where direction of dynamics and null-cline of *u* and *v* are also shown (“A” for the cell of Figure 3A and “B” for that of Figure 3D). The data is sampled in every grid from the branch terminals. *v* increases linearly with *u* from the cell boundary to the interior of the cell. Solid lines and dashed lines represent *f*(*u*,*v*) = 0 and *g*(*u*,*v*) = 0, respectively. We have found that an elongation speed is about twice slower in rugged dendrites than in well-aligned ones (compare Videos S1 and S3). The difference in the positioning of the *u* − *v* values relative to the isoclines may potentially explain the difference in a velocity of pattern formation (our unpublished data).

In the all cells examined, *u* and *v* exhibited a linear relationship at the growing tip of dendrite (Figure 4A for smooth branches and Figure 4B for rugged ones). Starting from *u* = 0 at the distal margin of dendrite, *u* should increase with time and it is observed as spatial change in *u* from distal to more-proximal parts of dendritic terminals. In contrast, the spatial change in *v* cannot be explained by reaction dynamics: for instance, in a case of Figure 4A, *u* and *v* should increase and decrease, respectively, according to vector field. Nevertheless, the supply of the suppressor from proximal dendrites via its diffusion seems to counteract actions of reaction functions, resulting in the increase of *v* in the proximal direction. Thus, most likely diffusion plays an essential role in determining the dynamics of the suppressor at dendritic tips.

### Classical Turing Condition and Distinctive Dendritic Patterns

As described below, we conducted numerical analysis to examine the generality of our cell compartment model and to determine the conditions for growth of dendrites that could be common to various types of neurons.

**Figure 5:**
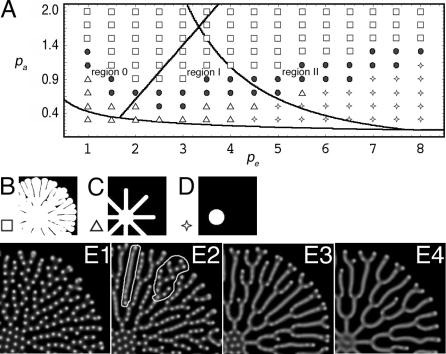
Parameter-Dependency of the Pattern Formation (A–D) Searches for parameter values for dendritic branch formation on a (*p_e_* − *p_a_*)-plane. The fixed parameters were *p_b_* = 0.8, *d* = 30.0, *p_h_* = 1.0, *Tr* = 1.0, *A*_max_ = 30.0, *R* = 0.004, and *γ* = 625. Total calculation time was 4 × 10^5^ steps. (A) Closed circle: dendritic pattern; square: wide branches; triangle: no second-order branching; and star: no growth. Examples of non-dendritic patterns of square, triangle, and star are shown in (B) (*p_a_* = 2.1 and *p_e_* = 8.0), (C) *p_a_* = 0.5 and *p_e_* = 4.0), and (D) (D (*p_a_* = 0.7 and *p_e_* = 6.5), respectively. Region I satisfies conditions of Turing diffusion-induced instability described by Equations 6a–6d, whereas region II satisfies Equations 6a–6c, but not Equation 6d and region 0 satisfies Equations 6b–6d, but not Equation 6a. In region I, spatially periodic patterns appear in a conventional RD model, whereas homogenous patterns are stable in region II. (E1–E4) Distributions of the activator that were obtained at different coordinates in the phase diagram (A). *p_a_* = 0.7 for all panels; and *p_e_* = 3.0 (E1), 3.5 (E2), 4.0 (E3), and 4.5 (E4). The distribution of the activator changes from a punctate pattern (E1) to a more continuous pattern (E4). (E2) A punctate distribution of the activator in a branch-rich region (enclosed area at right) and a more continuous one in a branch-less region (enclosed area at left).

We calculated the cell compartment model by using different parameter sets of reactions between the activator and the suppressor, and searched for those by which dendritic patterns were successfully generated (Figure 5A). We defined a dendritic pattern by the following two conditions: first, cellular extensions bifurcated. Second, the density of dendrites was less than a criteria value. Typical examples of patterns violating either of these conditions are shown in Figure 5B–5D. This analysis clearly shows that dendritic patterns could be generated in a large parameter region (closed circles in Figure 5A), and so formation of dendritic patterns in our model does not appear to depend on particular parameter sets.

As explained before, our model produced two different types of patterns: the well-aligned smooth pattern, in which the activator is continuously distributed (Figure 3A) and the poorly aligned rugged pattern, in which punctate distribution of the activator is seen (Figure 3D). Those patterns shown in Figure 3 are two extreme examples; and intermediate patterns could be generated, depending on parameters employed. Interestingly, our numerical analysis revealed a correlation between Turing instability [23] and the distinctive shape of dendritic patterns. Turing instability, a widely applied theory of pattern formation, indicates an ability of chemical (in this case, activator–suppressor) interactions to develop spatially periodic patterns. The condition of chemical reaction dynamics for Turing instability was addressed by considering the two-variable (*u* and *v*) dynamics in the uncompartmentalized 2D space (designated as no compartment model, that is, a conventional RD model), and then by numerically calculating a parameter region for potential Turing instability in the no-compartment model (see Equations A3a–A3d in the section “Conditions for Turing diffusion-induced instability” and region I in Figure 5A) [26]. We used typical values for other parameters such as *p_b_* because changing the *p_b_* value did not significantly alter the shape or the size of region I (unpublished data). The results of this analysis clearly showed that relatively rugged patterns were obtained by using the condition that satisfied Turing instability (region I in Figure 5A); on the other hand, better-aligned patterns were obtained by using the condition that did not satisfy it (region II in Figure 5A). Therefore, it is suggested that the difference in two typical dendritic patterns obtained in our computer simulation stems from whether chemical dynamics in themselves are able to develop spatially periodic patterns or not.

Furthermore, we noticed that the shape of dendrites reflected the intracellular distribution of the activator. From bottom-left to top-right of the (*p_e_* − *p_a_*) space (Figure 5A), the dendrite morphology became smoother; and distribution of the activator changed from punctate in nature to more continuous (Figure 5E1-5E4). Continuity in the activator distribution seems to strongly depend on the shape of local branches (Figure 5E2). Even within the same cell, the local distribution of the activator was punctate in branch-rich regions (e.g., right-enclosed branches in Figure 5E2), whereas it was more continuous in branchless regions (e.g., left-enclosed branch in Figure 5E2). Co-existence of two distinctive types of distributions, punctuate and continuous, in a single cell suggests that these two types of distributions are locally stable structures.

The above-mentioned analysis also indicated that the condition for developing dendritic patterns did not entirely cover region I. In addition, it is of particular interest that spatially non-homogeneous dendritic patterns were generated in region II, in which homogeneous distribution at the steady state should be stable in the two-variable (*u* and *v*) dynamics (see Discussion for details). Most likely this discrepancy of conditions for pattern formation in the cell compartment model and the no compartment one originates from the structure of cell and the feedback between the chemical reaction and cell growth in the model.

### Conditions for Dendritic Patterning and those for Dot Pattern Generation

We further examined the relationship between our model and the Turing system. In general, the Turing system develops dot, stripe, or reverse-dot patterns in the 2D space, depending on parameters (e.g., the distance from the equilibrium point to the upper limitation of activator [*A_max_*]) [28]. So we explored whether or not the conditions for dendritic pattern formation were related to the property of the Turing system to generate either a dot, stripe, or reverse-dot pattern.

**Figure 6:**
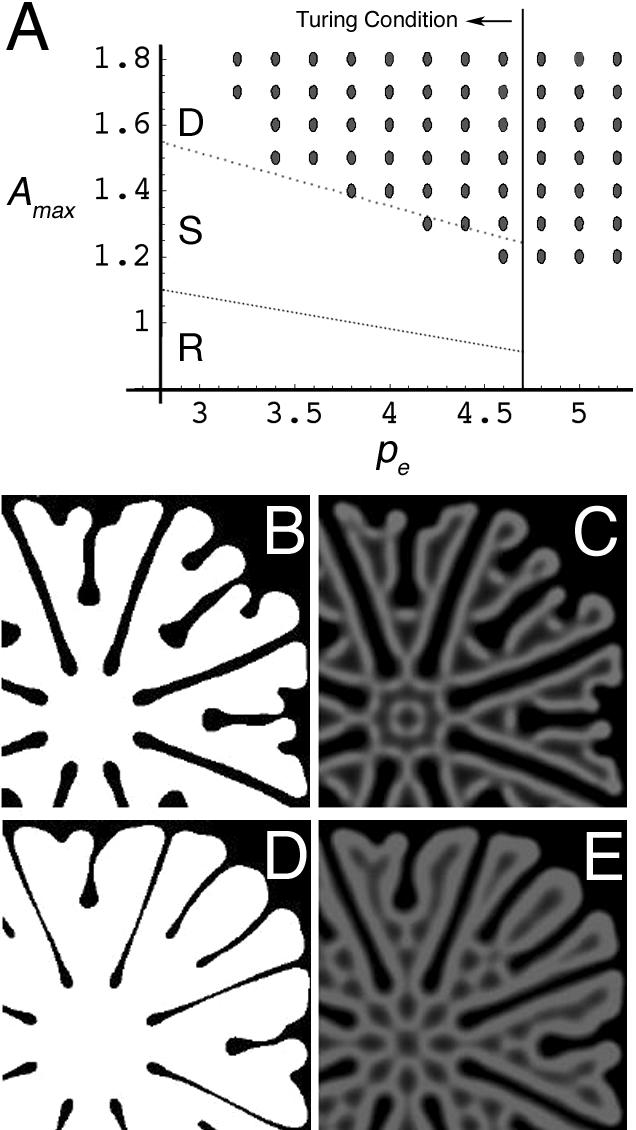
Comparative Analysis of Conditions for Dendritic Pattern Formation and Those for Dot, Stripe, and Reverse-Dot Pattern Generation (A) Condition of parameters for neuronal branching and Turing condition on a (*p_e_* − *A*_max_)-plane. Simulations of the no-compartment model showed that dot, stripe, and reverse-dot domains are mapped to different parameter regions (D, S, and R, respectively); and dotted lines roughly represent boundaries between the domains. Closed circles: dendritic patterns obtained by our cell-compartment model. The fixed parameters were *p_a_* = 0.7, *p_b_* = 0.8, *d* = 30.0, *p_h_* = 1.0, *R* = 0.004, and *γ* = 625. We used *Tr* = 0.95 (when *A*_max_ = 1.0), *Tr* = 0.85 (when *A*_max_ = 0.9), and *Tr* = 1.0 (otherwise). Total calculation time was 4 × 10^5^ steps. (B–E) Typical examples in the stripe domain (B,C): *p_e_* = 4.0, *A*_max_ = 1.1, and *Tr* = 1.0, and in the reverse-dot domain (D,E): *p_e_* = 3.6 and *Tr* = 0.95. Dendrites (B,D) and the distribution of the activator (C,E) are shown.

By changing the upper limitation of activator (*A_max_*) in the no-compartment model, we drew a phase diagram, in which each dot, stripe, and reverse-dot pattern was mapped to a different parameter region (Figure 6A). Subsequently we searched for parameter sets that developed dendritic patterns in the cell compartment model (circles in Figure 6A); and the results of this analysis indicated that dendritic patterns were obtained mostly in the dot domain (D in Figure 6A). Therefore the punctate distribution of the activator in rugged dendrites (Figure 3E) can be interpreted as the typical dot pattern of the conventional RD system being generated inside of the cell compartment. Dendritic patterns were not obtained in most of the stripe or reverse-dot domains (S or R in Figure 6A). Computer simulation with parameter settings in the stripe or reverse-dot domains generated patterns, which did not resemble the shape of dendritic arbors of real neurons (Figure 6B–6E). If conditions for Turing instability were not satisfied, dendritic pattern was produced in a parameter region adjacent to the dot domain. These results are consistent with an intuitive understanding of the process of dendritic pattern formation; that is, dendrites grow in pursuit of a track of locally activated molecular complexes for branching. In this sense, a punctate or terminally dense distribution of activator is favored, whereas the stripe or reverse-dot one is not.

### An Analysis of Other Dynamics

It is worth evaluating whether the results of this study are specific to a particular dynamics or if they represent more general properties of the RD system. For that purpose, we tested several different forms of reaction terms and one of them was as given below:

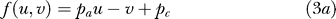


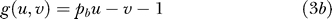



**Figure 7:**
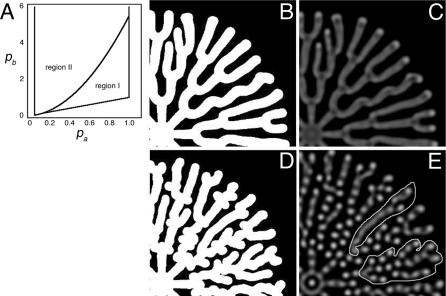
Patterns Generated by the Linear Dynamics (A) Region I indicates a parameter region on a (*p_a_* − *p_b_*)-plane that satisfies the classical Turing condition. *p_c_* does not appear in the Turing condition. Region II is as described in the legend of Figure 5. (B,C) and (D,E) Examples of patterns generated in region II and region I, respectively. Magnified images of dendrites (B,D) and those of activator distributions (C,E). Parameter values were *p_a_* = 0.5, *p_b_* = 2.5, and *p_c_* = 0.16 (B,C) and *p_a_* = 0.6, *p_b_* = 2.9, and *p_c_* = 0.2 (D,E). Other parameters were the same as in Figure 3A, except for *A*_max_ = 10.0 and *Tr* = 0.75. Conditions of simulation were as described in the legend of Figure 3.

Parameter settings for potential Turing instability in the linear dynamics described by Equations 3a and 3b were determined and plotted (region I in Figure 7A) as in Figure 5A.

**Figure 8:**
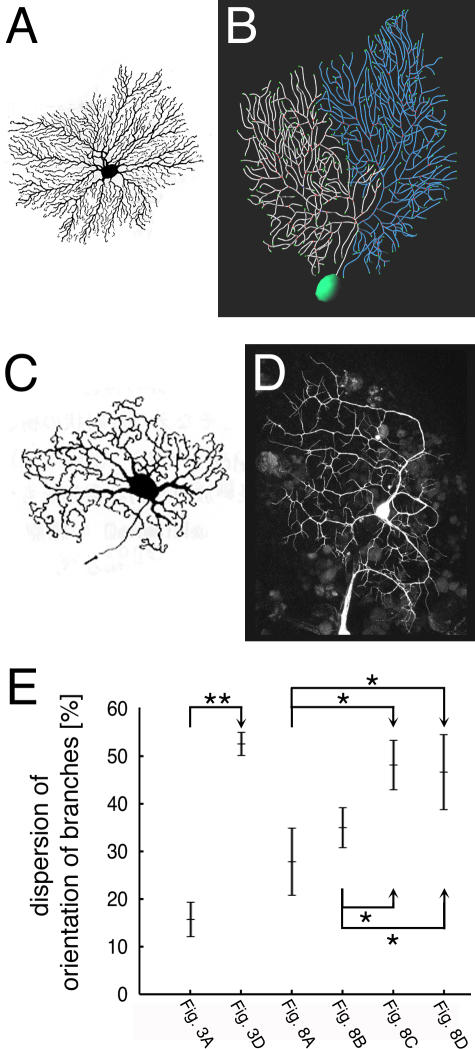
Two Distinctive Branching Patterns of Real Neurons (A,B) Smooth and well-aligned type. A neuron in thalamic nuclei in monkey (A) and Purkinje cell at postnatal day 25 (B). (C,D) Rugged and less-aligned type. A neuron of inferior olivary complex of monkey (C) and a remodeled class I da neuron at a pupal stage, which was visualized with *ppk*-*GAL4 UAS-mCD8::GFP* [49] (D). Images in (A and C) were taken from [50]. (E) Quantification of DOB in the generated patterns in our computer simulation. Data are presented as the means ± SD. A single asterisk indicates *p* < 0.01 (*t*-test), and double asterisks indicate *p* < 0.001 (*t*-test).

Parameter dependency of dendritic pattern formation was examined, and we found that dendritic patterns were generated in both outside and inside of region I (Figure 7B and 7D, respectively). Therefore, classical Turing conditions were not necessary or sufficient for dendritic pattern formation in this linear dynamics, either. Furthermore, whether the function was linear or non-linear, the activator distribution well-correlated with the shape of branches (Figure 7C and 7E; compare them to Figure 5E); and dendritic patterns were generated preferentially in the dot domain, but not in the stripe or reverse-dot domain (unpublished data). Collectively, all of the results suggest that a wide range of parameter settings and different dynamics of chemical reactants allow development of dendritic patterns in the cell compartment model.

### Morphological Measures for Predicting In Vivo Distribution of the Activator

Finally we found that our cell compartment model provides a prediction for future experiments. As described before, the numerical simulation of the model unraveled a strong correlation between shapes of dendrite and distributions of the activator (Figure 5E and Figure 7E). We noticed that dendritic trees of some real neurons were reminiscent of those of the smooth type in our computer simulation (Figure 8A and 8B) and that terminal branches of some other real neurons were less aligned (Figure 8C and 8D). Accordingly, if the developmental machinery proposed by this study is actually functioning in vivo, the intracellular distribution of the hypothetical activator could be predicted on the basis of the morphological features of dendrites. More specifically, the distribution of the activator may be terminally dense in neurons of the smooth type and punctate in the rugged type (for instance, Figure 8A and 8B and Figure 8C and 8D, respectively).

**Figure 9:**
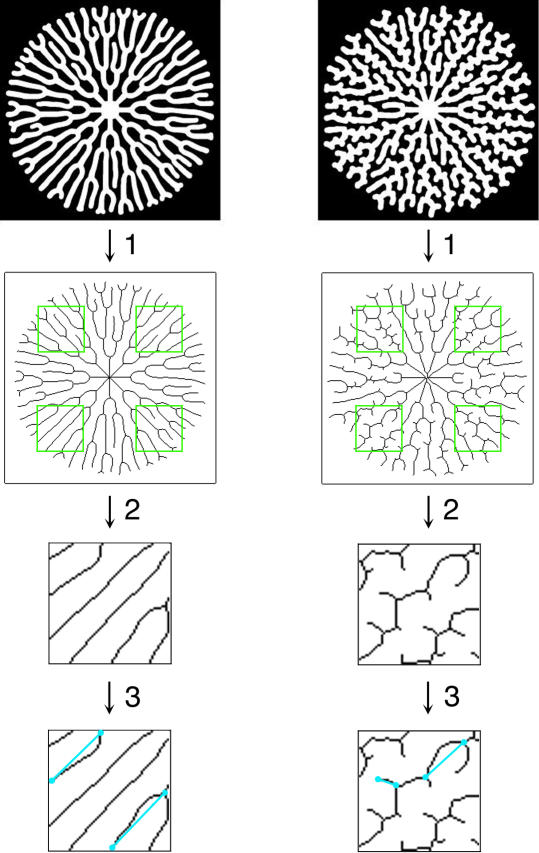
Procedures for Quantification of DOB Step 1: Skeletonize images with ImageJ. Step 2: Crop four pairs of squares. Step 3: Approximate each dendritic segment between the two branching points by a line segment connecting those points. Measure the angle of a branch segment with respect to the horizontal direction. Repeat measurement for all segments in each local area and calculate the coefficient of variation, DOB.

To support the validity of our prediction, we set a quantitative measure called a “dispersion of orientation of branches” (DOB) to characterize dendrite morphology. DOB is the coefficient of variation of directions of branch segments in each local region of dendritic trees (Figure 9 and Materials and Methods); hence the smaller is the DOB, the better-aligned are the local branches. Quantification of the DOB for the smooth and rugged types of the obtained patterns in our computer simulation confirmed that it was significantly smaller in the former type (double asterisks in Figure 8E). We next quantified the DOB for real neurons and found that values for the smooth type (Figure 8A and 8B) were significantly smaller than those for the less-aligned type (Figure 8C and 8D; asterisks in Figure 8E). These results suggest that geometry of real neurons may also be characterized by DOB and that we can use DOB as a morphological measure for predicting the intracellular distribution of the activator in vivo.

## Discussion

### Self-organizing Mechanism in Dendrite Pattern Formation

In this study, we developed the first mathematical model that sheds light on autonomous pattern formation of neuronal dendrites. The cell compartment model, which is based on the experimentally verified dendro-dendritic interaction, autonomously develops dendritic elongation and branching. It should be noted that dendritic patterns are defined not only by the numerical parameters such as the terminal number, but also by other properties such as mutual avoidance. Our model places emphasis on the latter aspects of the space-filling dendrites, which are difficult to characterize by quantitative measures, and indeed qualitatively recaptures developmental regulation of the space-filling dendritic patterns. Collectively, we believe that this study offers a new concept in developmental biology, a self-organizing mechanism in neuronal dendrite pattern formation.

Many of the previous models assumed that elongation and branching of dendrites are controlled by probability functions, in which each parameter separately codes individual growth rules such as degree- or segment length- dependent rate of elongation and/or branching [19,20]. In contrast, dendritic patterns are autonomously generated without embedding different parameters to control each branching frequency, branch angle, and self-avoidance of dendrites in our model. Considering that we are presently far from understanding the entirety of the molecular mechanisms of chemical reactions occurring in vivo, the high performance of the proposed system obtained with diverse forms of reaction function takes on significance, because it may support a future application of the model to the dendritogenesis of a whole variety of real neurons.

### Instability of Chemical Dynamics and That of Cell Boundary Contribute to Dendritic Pattern Formation

Our numerical analysis showed that generation of dot patterns of the activator in rugged dendrites could be attributed to a property of chemical dynamics, which is supported by Turing instability. On the other hand, classical Turing diffusion-induced instability alone cannot give us a comprehensive explanation of the pattern formation in our model, because dendritic patterns were successfully developed even when the spatially homogeneous pattern at the steady state of chemical reaction dynamics was stable. We think that the compartmentalized structure in our model may increase instability of the dynamics of the cell growth. Actually, it was shown both in experiments and in computer simulation that a straight interface could become unstable to make complex spatial patterns in certain bistable dynamics [27,29]. Hence, analyzing the model based on the idea of front instability may be one way to understand the behavior of our model. From a viewpoint of experimental biology, these results suggest that simultaneous, high-resolution imaging analyses on molecular interactions and plasma membrane dynamics would be informative.

### Distribution of the Activator In Vivo

We introduced new criteria to categorize patterns of dendrites in real neurons and to predict the intracellular distribution of potential molecular complexes for dendrite growth. Two distinctive dendritic patterns were found in both computer-simulated and real neurons, and it is suggested that the distribution of the activator is characteristic of the shape of branches. Further advances in our understanding of the molecular mechanisms involved in dendrite development are required to address whether the prediction from our cell compartment model is valid or not. Yet, there are a couple of interesting observations that may indicate periodicity in dendrites of real neurons. For instance, Golgi apparatus is distributed in a punctate manner in da neurons and pyramidal neurons [30–32]; and its localizations at branch points are important for branch formation. In addition, staining for microtubule-associated protein 2 in the absence of detergents reveals that regions of high signal intensity are found in a spatially periodic manner along dendrites and that dendritic branch points are preferentially associated with these regions [33]. It would be interesting to review these observations in the perspective of our model.

### Enlarged Editions of the Cell Compartment Model

Our cell compartment model is a simplified version of dendrite growth in vivo, and new elements can be installed depending on needs or researchers' interests. For instance, although generated patterns in the present model are highly homogeneous, less homogeneous patterns could be obtained if stochastic aspects or noise are strengthened (for example, by fluctuating *Tr* along dendritic branches). It is also interesting to extend our model to include activity-dependent processes, such as synaptotropic dendrite growth [34,35] and refinement of pre-existing branches during late stages of development [36,37]. Furthermore, we are now trying to reproduce development of non–space-filling type dendrites, which are anisotropic in terms of the direction of elongation and inhomogeneous in terms of coverage of a field, by incorporating a guidance mechanism and/or an RD system of intracellular activator and suppressor. Although we should bear in mind that overlaying these additional features could modify the properties of the system, we hope that combination of biochemical experiments with enlarged editions of this mathematical model may clarify the comprehensive logic underlying neuronal dendrite development.

### Insights to Other Types of Branching Morphogenesis

Colony formation by Bacillus subtilis is a well-known example of dendritic patterning in biology. Bacillus subtilis generates distinctive colony patterns depending on the substrate softness and nutrient concentration [38], and formation of most of the colony patterns was well-reproduced by RD models [39,40] and a cell automaton model [41]. Similarity between neuronal dendrites and bacteria colonies is found not only in terms of their morphology, but also with respect to repulsive behaviors; i.e., when two colonies are in close proximity, they avoid each other just as do space-filling neurons [42]. In addition, interesting parallels can be also found between dendrite development and other branching morphogenesis such as coral [43], vertebrate lung [44], and trachea of *Drosophila* [45]. These systems accomplish physiological functions that can be regarded as similar to space-filling dendrites. For instance, trachea must elaborate its branches to deliver oxygen to the whole body. Mathematical models for these pattern formations have been proposed [43,44,46,47], and it is suggested that branching morphogenesis in general can be understood as the following: a part of the structure that happens to sprout due to some fluctuation locally speeds up its growth and eventually develops a visible branch. We observed a similar behavior of dendrites in our computer simulation. Furthermore, recent works revealed the molecular basis of lateral inhibition between the neighboring lung epithelium and between growing tips of trachea that may correspond to long-range inhibitory dendro-dendritic interactions in the development of space-filling dendrites [44–46]. Therefore, our model on neurite formation would be potentially informative in understanding the above-mentioned branching morphogenesis.

Despite the afore-mentioned similarities, there is one big difference between bacteria colony models and ours. The former relies on non-linearity in diffusion and reaction function for pattern formation [39]. On the other hands, dendritc growth in our model does not require such non-linearity (Figure 7). It might be that unambiguous boundary of the cell in our model plays an equivalent role to non-linear diffusion terms in bacteria colony models. Taking advantage of the fewer constraints in chemical dynamics in our model, we addressed the relationship between Turing instability and biological branching morphogenesis. Other branching morphogenesis might obey the conditions that were clarified in this study. Again, generality of the proposed mechanism would be significant for testing this possibility in other systems of interest.

## Materials and Methods

### Numerical analysis.

To calculate the model, we used the finite difference method, a simple explicit scheme. The simulation starts from a small cell body. The initial value of the activator is 0.5± small random deviations in each position inside of the cell body and 0 in other places, whereas the value of the suppressor is 0.1± small random deviations in the cell body and 0 otherwise. Changes in initial conditions of the activator or the suppressor affected the results only slightly. Small noise was added to the diffusion coefficient of the activator in every calculation step to cancel the anisotropy of the grid in numerical simulation.

### Quantification of dispersion of orientation of branches.

Image processing and measurement were done with ImageJ. First, we superimposed a square on individual dendritic trees (those in Figure 3A and 3D and Figure 8A–8D; see also Figure 9). The size of each square was normalized to that of the entire dendritic tree (the size of the tree was defined as that of a polygon connecting dendritic tips). As for the obtained patterns in computer simulation (those in Figure 3A and 3D), we skeletonized them and sampled four pairs of squares that were located at the same coordinates (“1” and “2” in Figure 9). Each branch segment was approximated by a line segment connecting two edges of the branch segment (“3” in Figure 9). We measured the angle of the line segment with respect to the horizontal direction, repeated measurement for all segments in each small square, and calculated the coefficient of variation, which we called the DOB. Average values of DOB for each dendritic tree are shown with means ± SD in Figure 8E.

### Imaging collection of Dendritic trees.

Imaging and single cell labeling of *Drosophila* sensory neurons were done as described [11,13,48]. Strains used were *NP7028 UAS-mCD8::GFP* [11], *ppk*-*GAL4 UAS-mCD8::GFP* [49], *elav-GAL4 UAS-mCD8::GFP hsFLP*, *tub-Gal80 FRT40A*, and *FRT40A* [13].

### Original equations.

Original equations of the activator-suppressor model were as follows:

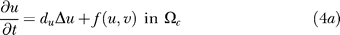


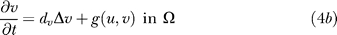


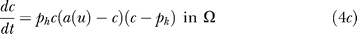
where *u* and *v* are the concentration of the activator and that of the suppressor, respectively. *d_u_* and *d_v_* are diffusion coefficients. Original chemical reaction terms were:

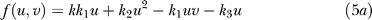


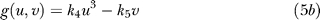



We non-dimensionalized Equations 4a–4c and Equations 5a–5b to obtain Equations 1a–1c and Equations 2a–2b.

### *R* value.

The value of *R* determines the thickness of the branches as expected. Smaller *R* resulted in thinner branches, thus finer patterns. However, there seems to be a minimum value of *R* to support dendrite growth. The minimum value may be necessary to produce a new spot of the activator, which is separated from the pre-existing spot, in the vicinity of the cell boundary. We confirmed that the minimum value of *R* was independent of the spatial grid size in numerical simulation, and thus the above results are not an artifact of numerical simulation. So we used *R* = 0.004, which gave the finest dendritic patterns (*R* = 0.0041 yielded nearly equal results to those obtained with *R* = 0.004).

### Conditions for Turing diffusion-induced instability.

Conditions for Turing diffusion-induced instability [23] are the following:

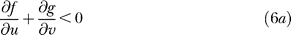


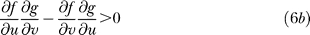


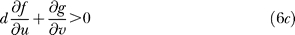


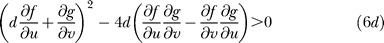
where the partial derivatives of *f* and *g* are evaluated at the steady state (*u*_0_,*v*_0_) which satisfies f(*u*_0_,*v*_0_) = 0 and g(*u*_0_,*v*_0_) = 0 [26]. Equations 6a and 6b describe conditions for a stable equilibrium point in the absence of diffusion. Equations 6c and 6d describe conditions for an unstable periodic solution in the presence of diffusion.

## Supporting Information

Video S1 Formation of a Well-Aligned Dendritic Pattern One frame of this movie is shown in Figure 3A.
(84 KB MOV)

Video S2 Continuous, but Terminally Enriched Distribution of the Activator During Dendritic Growth Shown in Video S1
Five frames of this movie are shown in Figure 3B.
(24 KB MOV)

Video S3 Formation of a Rugged Dendritic Pattern One frame of this movie is shown in Figure 3D.
(189 KB MOV)

Video S4 Punctate Distribution of the Activator During Dendritic Growth Shown in Video S3
Five frames of this movie are shown in Figure 3E.
(71 KB MOV)

## Abbreviations

da: dendritic arborization
DOB: dispersion of orientation of branches
